# Decreased stage migration rate of early gastric cancer with a new reconstruction algorithm using dual-energy CT images: a preliminary study

**DOI:** 10.1007/s00330-016-4442-z

**Published:** 2016-06-08

**Authors:** Cen Shi, Huan Zhang, Jing Yan, Baisong Wang, Lianjun Du, Zilai Pan, Fuhua Yan

**Affiliations:** 1 Department of Radiology, Ruijin Hospital, Shanghai Jiao Tong University School of Medicine, No.197, Ruijin 2nd Road, Shanghai, 200025 China; 2 Department of Radiology, the First Affiliated Hospital of Soochow University, 188 Shizi Road, Suzhou, 215006 China; 3 Siemens Medical System, Shanghai, 201318 China; 4 Department of biological statistics, Shanghai Jiao Tong University School of Medicine, Shanghai, 200025 China

**Keywords:** Early gastric cancer, Dual-energy Computed Tomography, Monoenergetic images, Advanced monoenergetic images, Polyenergetic images

## Abstract

**Objectives:**

To evaluate the potential value of advanced monoenergetic images (AMEIs) on early gastric cancer (EGC) using dual-energy CT (DECT).

**Methods:**

31 EGC patients (19 men, 12 women; age range, 38–81 years; mean age, 57.19 years) were retrospectively enrolled in this study. Conventionally reconstructed polyenergetic images (PEIs) at 120 kV and virtual monoenergetic images (MEIs) and AMEIs at six different kiloelectron volt (keV) levels (from 40 to 90 keV) were evaluated from the 100 and Sn 140 kV dual energy image data, respectively. The visibility and stage migration of EGC for all three image data sets were evaluated and statistically analyzed. The objective and subjective image qualities were also evaluated.

**Results:**

AMEIs at 40 keV showed the best visibility (80.7 %) and the lowest stage migration (35.5 %) for EGC. The stage migration for AMEIs at 40 keV was significantly lower than that for PEIs (*p* = 0.026). AMEIs at 40 keV had statistically higher CNR in the arterial and portal phases, gastric-specific diagnostic performance and visual sharpness compared with other AMEIs, MEIs and PEIs (all *p* < 0.05).

**Conclusions:**

AMEIs at 40 keV with MPR increase the CNR of EGC and thus potentially lower the stage migration of EGC.

***Key Points*:**

• *AMEIs benefits from the recombination of low-keV images and medium energies.*

• *AMEIs could receive better CNR results than MEIs.*

• *AMEIs at 40 keV potentially lower the stage migration of EGC.*

**Electronic supplementary material:**

The online version of this article (doi:10.1007/s00330-016-4442-z) contains supplementary material, which is available to authorized users.

## Introduction

In clinical, therapeutic approach decisions depend on accurate preoperative staging. Early gastric cancer (EGC) can be treated with more limited surgeries, such as endoscopic mucosal resection (EMR) and laparoscopic surgery [1–3]. Preoperative chemotherapy or radiation therapy is usually recommended for advanced gastric cancer (AGC) to downstage the tumour and increase the chance for curative resection [4]. Currently, two-dimensional (2D) multi-detector computed tomography (MDCT) imaging using multiplanar reconstruction (MPR) has been widely used for the preoperative staging of gastric cancer because of the ability to detect the depth of tumour invasion and the presence or absence of metastasis [5–8]. However, its detection rates of EGC are unsatisfactory. For example, Makino et al. reported a detection rate of only 19 % using MDCT with MPR [6].

In the evaluation of EGC, the use of various three-dimensional (3D) reconstruction techniques, such as virtual gastroscopy, has led to improved diagnostic performance compared with conventional 2D imaging [9–13]. Nevertheless, one main disadvantage of 3D techniques is how time consuming they are. Although greater computer processing power makes more rapid reconstructions possible, the entire procedure takes approximately 20–30 minutes per patient [10]. Compared with 3D technologies, 2D imaging is more straightforward.

Dual-energy CT (DECT) can provide material decomposition information, especially iodine concentrations which could be used to analyse tumour perfusion and detect small iodine content lesions [14, 15]. DECT can also create “virtual” monochromatic images at a range of keV. Most previous studies have focused on CT angiography, which is significantly affected by the lower keV required to obtain image qualities with acceptable CNR and signal-to-noise ratio (SNR) or lower amount of contrast medium [16–18]. Few studies have investigated the effect of lower keV on tissue applications, particularly in hollow viscera, such as the stomach, because the enhancement on their walls is less concentrated than that observed in solid organ (e.g., liver). In addition, because the image noise usually increases even more than the iodine contrast at lower energy levels due to the absorption of lower-energy photons, the CNR might decrease at low keV. Thus, few applications employ lower keV (e.g., 40 keV or 50 keV) while simultaneously obtaining higher contrast and lower noise [19, 20].

A new prototype algorithm has been developed to calculate advanced monoenergetic images (AMEIs) (Dual energy Mono+, syngo IPIPE, Siemens Healthcare, Forchheim, Germany). As the prototype software has not been available for commercial use, it has been used for research purposes only in our institution. The purpose of this study was to explore the potential value of AMEIs in EGC.

## Materials and methods

### Patients

**Fig. 1:**
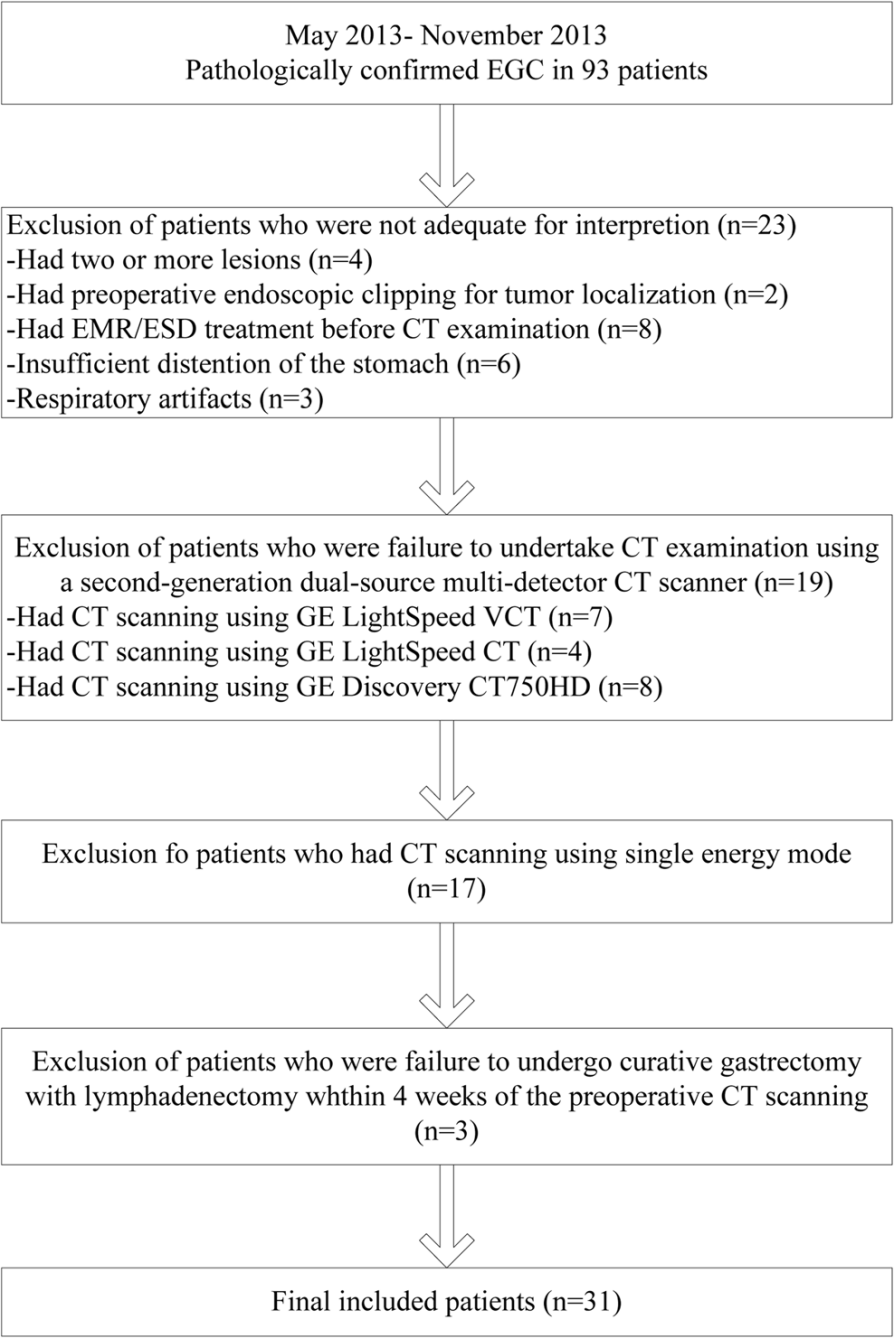
Flowchart of patient selection

This retrospective study was approved by our institutional review board, and the requirement for informed consent was waived. From May to December 2013, 93 consecutive patients were pathologically confirmed to have EGC in our institution. A flowchart of the selection of these patients is presented in Fig. 1. The final study population consisted of 31 patients, including 19 men and 12 women, ranging in age from 38 to 81 years (mean ± standard deviation: 57.19 years ± 10.33). Mucosal tumours were found in 18 patients (58.06 %), and submucosal tumours in 13 (41.93 %), according to the Japanese Classification of Gastric Carcinoma [21].

### CT examination

All 31 patients underwent CT after overnight fasting to empty the stomach. Before CT examinations, each patient drank 1000–1500 ml of tap water and was injected with 20 mg of scopolamine; they then underwent contrast-enhanced dual-energy CT (Siemens SOMATOM Definition Flash, Siemens Medical Solutions, Forchheim, Germany). CT scans were acquired with the tube voltages at 100 and 140 kV with a tin filter (i.e., 100/Sn140 kV), using reference mAs values of 230 and 178, respectively. The collimator was 32 × 0.6 mm, and the pitch was 0.6. All acquisitions were obtained with real-time tube current modulation (CARE Dose 4D, Siemens Medical Solutions). To estimate the time to peak enhancement of the celiac trunk, 16 ml contrast was first injected as a test bolus. Then the main bolus (1.5 ml iopromide per kilogram of body weight, Ultravist 370; Schering, Berlin, Germany) was injected at a rate of 3 ml/s. Three phasic, contrast-enhanced, dual-energy CT scans were performed on each patient, which included an arterial phase (AP) (determined by the time to peak enhancement of the celiac trunk) covering the whole stomach, a portal venous phase (PP) (20 s after the AP), ranging from the diaphragmatic domes to the anal verge, and a delayed phase (DEP) (150 s after the administration of contrast agents), covering the whole stomach. The mean scan delay time of AP was 15.10 ± 6.710 seconds (range, 6–28 seconds after injection), and the mean scan delay time of PP was 35.06 ± 6.673 seconds (range, 26–48 seconds after injection). For radiation dose, the mean CTDvol and DLP, which includes all phases, were 34.8 ± 7.1 mGy and 1080.5 ± 336.9 mGy · cm, respectively.

The DE raw data were reconstructed using a kernel of D30f. Three different series of images were generated: 100 kV images, Sn140 kV images, and mixed 120 kV PEIs, with a linear blending technique using a slice-thickness ratio of 0.5. Low 100 kV and high Sn140 kV images were then transferred to the workstation (Dual energy Monoenergetic, syngo MMWP, version 2008A; Siemens Healthcare, Forchheim, Germany) to generate six data sets of MEIs in 10-keV intervals (40-90 keV). Low 100 kV and high Sn140 kV images were also transferred to a personal computer with the prototype software (Dual Energy Mono+, Syngo IPIPE, Siemens Healthcare, Forchheim, Germany) to generate the six data sets of AMEIs in Dicom format with the same keV levels in 10 seconds for each patient in each scan phase. Then, all images were imported to the workstation and MPR images were also reconstructed, which were interpreted on the diagnostic monitors by radiologists. As the prototype software has not been available for commercial use, it has been used for research purposes only in our institution.

### Image analysis

**Fig. 2:**
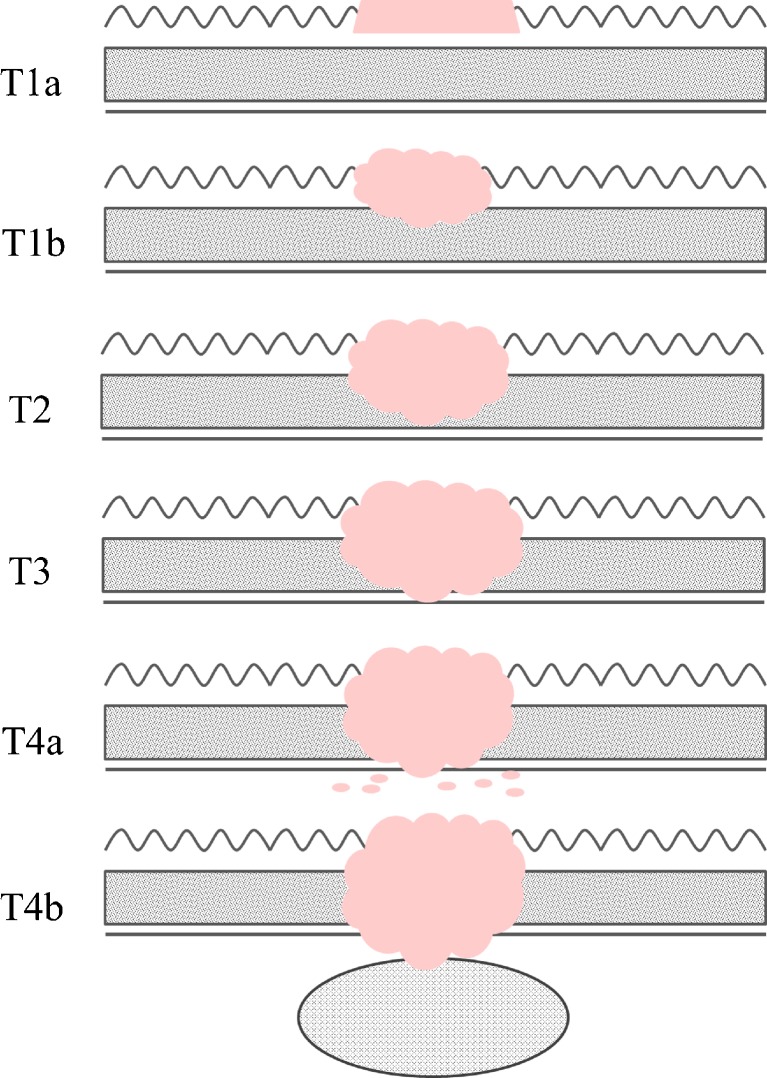
Pictorial examples for each stage presented by CT

All images were evaluated by two abdominal radiologists (L.J.D. and Z.L.P), both with 10 years of experience in gastrointestinal imaging, who were completely blinded to the surgical and histological findings (they were aware that the patients had histologically proven gastric cancers, but completely blinded to lesion location, size, macroscopic features, and stage of the gastric cancers). Differences in assessment were resolved by consensus. The PEIs, 40–90 keV MEIs and AMEIs were anonymized and randomly assigned case numbers from 1 to 403. All data sets were randomly divided into 13 groups with 31 series of images per group. The two radiologists interpreted one group of images each time. To minimize recall bias, each reading session was separated by one week. The visibility and T staging of the tumours were evaluated on each series of CT images. The definitions used for T staging were summarized in Table 1 [22] (Fig. 2). The radiologists recorded the locations and sizes of the tumours. MDCT and pathologic findings regarding the locations and sizes of the gastric cancers were correlated by a third abdominal radiologist (C.S.) with 3 years of clinical experience. When the tumour was in the same location on the CT images as the pathology specimen and the tumour size measured from the CT images was approximately the same as the pathologic measurement, the tumour was defined as visible. The rates of stage migration were calculated. Taking the pathological results as the reference standard, different numbers of patients may be incorrectly staged by different reconstruction algorithms. Therefore, the incorrectly staged patients, including the invisible patients and over-staged patients, were considered as stage migration.

**Table 1:** MDCT criteria for the tumour staging of gastric cancer

Stage (depth of invasion)	MDCT criteria
T1 (mucosa/submucosa)	Tumour shows enhancement and/or thickening of the inner mucosal layer, as compared to the adjacent normal mucosal layer, with an intact low-density-stripe layer (T1a) or disruption of the low-density-stripe layer (less than 50 % of the thickness) (T1b)
T2 (muscularis propria)	Disruption of the low-density-stripe layer (greater than 50 % of the thickness) is visualized without abutting on the outer, slightly high-attenuating layer
T3 (subserosa)	Discrimination between the enhancing gastric lesion and the outer layer is visually impossible, and a smooth outer margin of the outer layer or a few small linear strandings in the perigastric fat plane are visualized
T4 (serosa/adjacent structures)	An irregular or nodular outer margin of the outer layer and/or a dense band-like perigastric fat infiltration is visualized (T4a), or obliteration of the fat plane between the gastric lesion and the adjacent organs or direct invasion of the adjacent organs (T4b)

The two readers were asked to assess the contrast-to-noise ratio (CNR) of the lesion of each phase. Free-hand regions of interest (ROIs) were placed in the lesion and normal gastric wall to measure the attenuation values in Hounsfield units (HU). When the lesion was invisible, their attenuation was recorded as equal to the normal gastric wall. In addition, ROIs were placed in the psoas muscle to estimate the image noise. Subsequently, CNR was calculated using the following formula:

$$ \mathrm{C}\mathrm{N}\mathrm{R} = \left({\mathrm{HU}}_{\mathrm{lesion}}\hbox{-}\ {\mathrm{HU}}_{\mathrm{normal}}\right)/\ {\mathrm{noise}}_{\mathrm{muscle}} $$

The readers were also asked to assess gastric-specific diagnostic confidence using a 5-point scale (1 = undiagnostic; 2 = will potentially miss lesions; 3 = will likely not miss or mischaracterize lesions; 4 = most likely will identify all abnormalities; 5 = can easily detect all lesions). Visual sharpness was graded on a 5-point scale (1 = unacceptable; 2 = poor; 3 = equivocal; 4 = good; 5 = excellent). Image noise was rated on a 4-point scale (1 = less than usual; 2 = optima [routine] noise; 3 = increased noise, does not affect interpretation; 4 = increased noise affecting interpretation).

### Statistical analysis

Statistical analysis was performed using SPSS software (SPSS version 16.0, SPSS, Chicago, IL, USA). Continuous variables were expressed as the mean ± standard deviation. Ordinal variables are reported as median (range). Comparisons of all variables between MEIs, AMEIs and PEIs were performed. Comparisons of visibility and stage migration were performed using the McNemar test. Differences in CNR were estimated using a paired *t*-test. In addition, a Wilcoxon signed rank test was performed to compare gastric-specific diagnostic performance, image noise and visual sharpness. Box plots were used to visualize means, upper and lower extremes and upper and lower quartiles of CNR. *p* values were adjusted using the Adaptive False Discovery Rate method (SAS, version 9.2; SAS Institute, Gary, NC) as multiple comparisons were performed. A two-tailed *p* value of less than 0.05 was considered to indicate a statistically significant difference. To assess the degree of observer agreement, we used weighted kappa statistics. We considered a *k* value greater than 0.81 to be representative of almost perfect agreement and values of 0.61 to 0.80, 0.41 to 0.60, and less than 0.41 to be representative of substantial, moderate, and poor agreement, respectively.

## Results

### Pathology findings

EGCs can be divided into three macroscopic types [21]: I, protruding type; II, superficial type (IIa: elevated, IIb: flat, and IIc: depressed); and III, excavated type. According to the histological findings, two lesions were classified as protruding type, 19 lesions as superficial type (2 as IIa, 6 as IIb and 11 as IIc), and ten lesions as excavated type. By location, nine tumours occurred on the body, seven on the angle, and 15 on the antrum. The mean maximum diameter of the tumours was 1.74 ± 1.37 cm (range, 0.6–3.0 cm).

### Visibility and stage migration of the primary tumour

The visibility and stage migration values are summarized in Table 2. Visibility was significant higher for AMEIs at 40 keV (AM40 keV) compared with MEIs at 40 keV (M40 keV), 50 keV (M50 keV), and 60 keV (M60 keV) (*p* = 0.008, 0.008, and 0.045, respectively). There were no significant differences between AM40 keV and the other MEIs and PEIs (all *p* > 0.05). M40 keV and M50 keV showed significantly worse visibility than the other MEIs or PEIs, with the exception of M60 keV. The rate of stage migration was significantly lower for AM40 keV compared with other AMEIs, MEIs and PEIs (all *p* < 0.05), with the exception of AM50 keV, AM60 keV and AM70 keV (*p* = 0.250, 0.083 and 0.064, respectively). In addition, only AM40 keV showed a significantly lower stage migration compared with the PEIs (*p* = 0.026). (Details are provided in “Supplementary Information”.)

**Table 2:** Visibility, over-staging and stage migration of MEIs, AMEIs and PEIs

Group	cT0	cT1	cT2	cT3	cT4	Visibility	Over-staging	Stage migration
PEIs	11	12	5	3	0	20 (64.5 %)	8 (25.8 %)	19 (61.3 %)
AM40 keV	6	20	5	0	0	25 (80.7 %)	5 (16.1 %)	11 (35.5 %)
AM50 keV	7	17	7	0	0	24 (77.4 %)	7 (22.6 %)	14 (45.2 %)
AM60 keV	8	15	8	0	0	23 (74.2 %)	8 (25.8 %)	16 (51.6 %)
AM70 keV	9	14	7	1	0	22 (71.0 %)	8 (25.8 %)	17 (54.8 %)
AM80 keV	10	10	10	1	0	21 (67.7 %)	11 (35.5 %)	21 (67.7 %)
AM90 keV	10	10	10	1	0	21 (67.7 %)	13 (41.9 %)	21 (67.7 %)
M40 keV	19	0	5	7	0	12 (38.7 %)	12 (38.7 %)	31 (100.0 %)
M50 keV	18	0	8	5	0	13 (41.9 %)	13 (41.9 %)	31 (100.0 %)
M60 keV	13	5	10	3	0	18 (58.1 %)	13 (41.9 %)	26 (83.9 %)
M70 keV	10	10	7	4	0	21 (67.7 %)	11 (35.5 %)	21 (67.7 %)
M80 keV	10	9	9	3	0	21 (67.7 %)	12 (38.7 %)	22 (71.0 %)
M90 keV	10	9	8	4	0	21 (67.7 %)	12 (38.7 %)	22 (71.0 %)

**Fig. 3:**
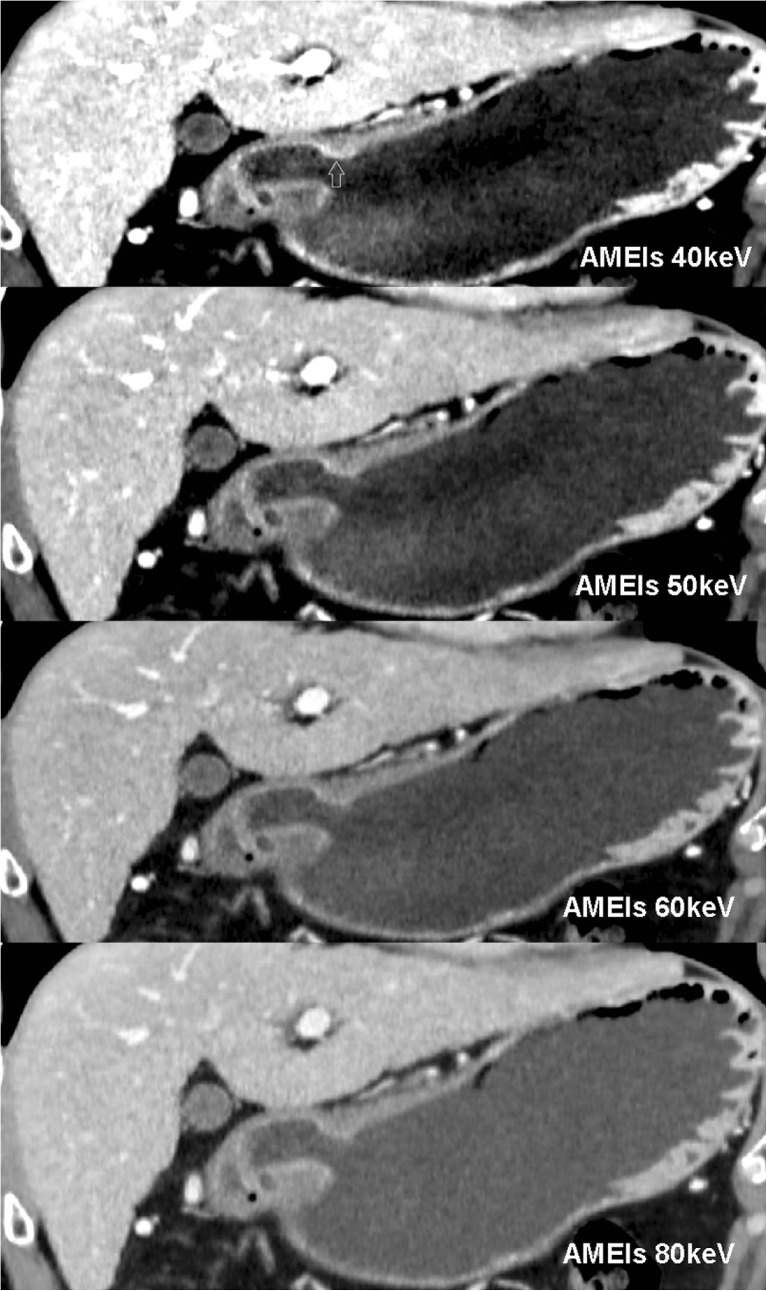
T1a cancer (54 yrs, male) in AM40, 50, 60, 80 keV. AM40 keV coronal image shows abnormal strong enhancement of the inner mucosal layer with an intact low-density-stripe layer (*arrow*) in the gastric angle in the portal phase. The lesion was classified as T1a by two reviewers. The lesion is not clear in AM50 keV and is invisible in either AM60 keV or AM80 keV

**Fig. 4:**
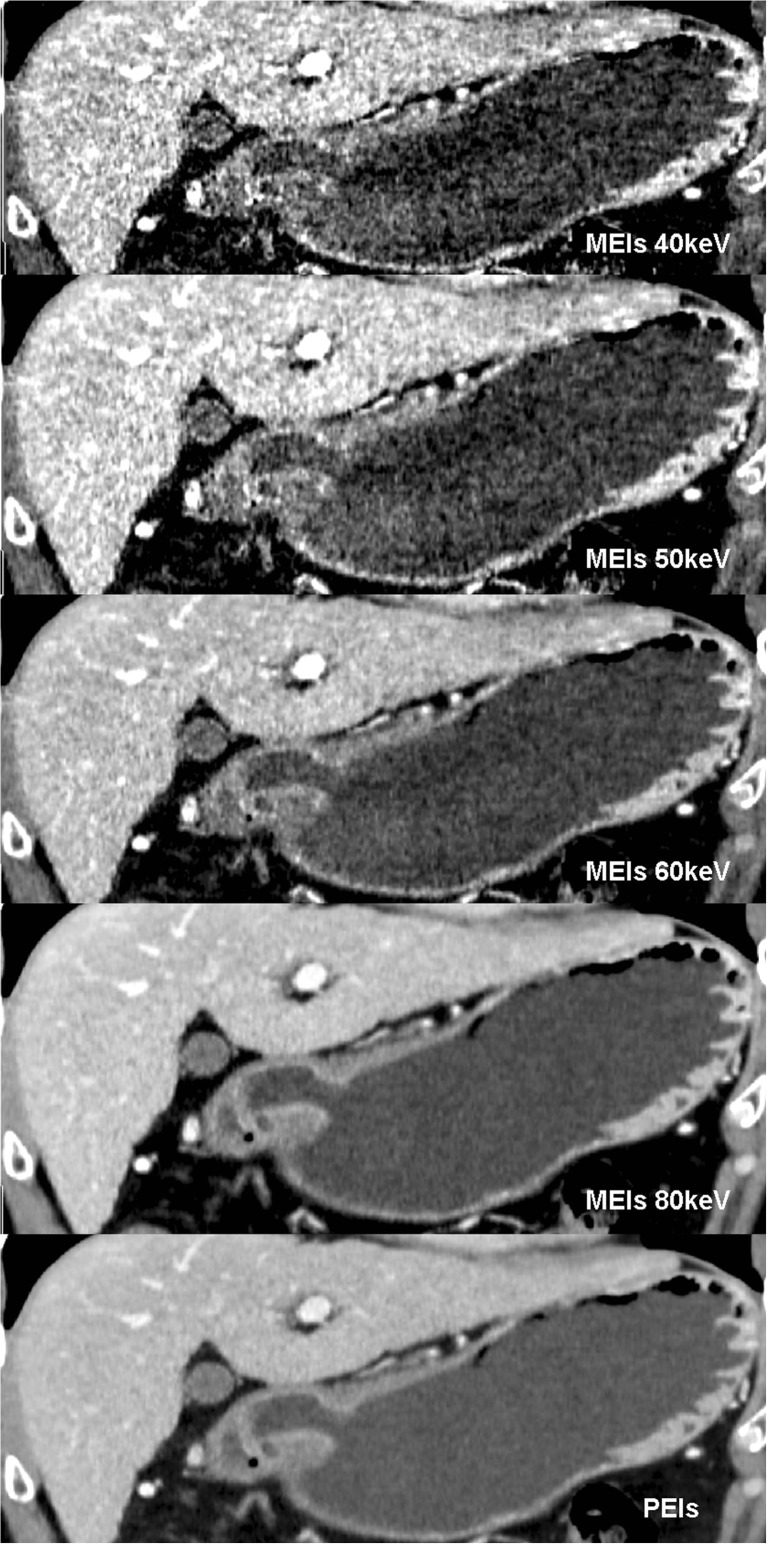
The same patient in Fig. 3 in M40, 50, 60, 80 keV and PEIs. The lesion is invisible in M40 keV, M50 keV because of the high image noise, which affects diagnosis. The lesion is also invisible in M60 keV, M80 keV and PEIs

**Fig. 5:**
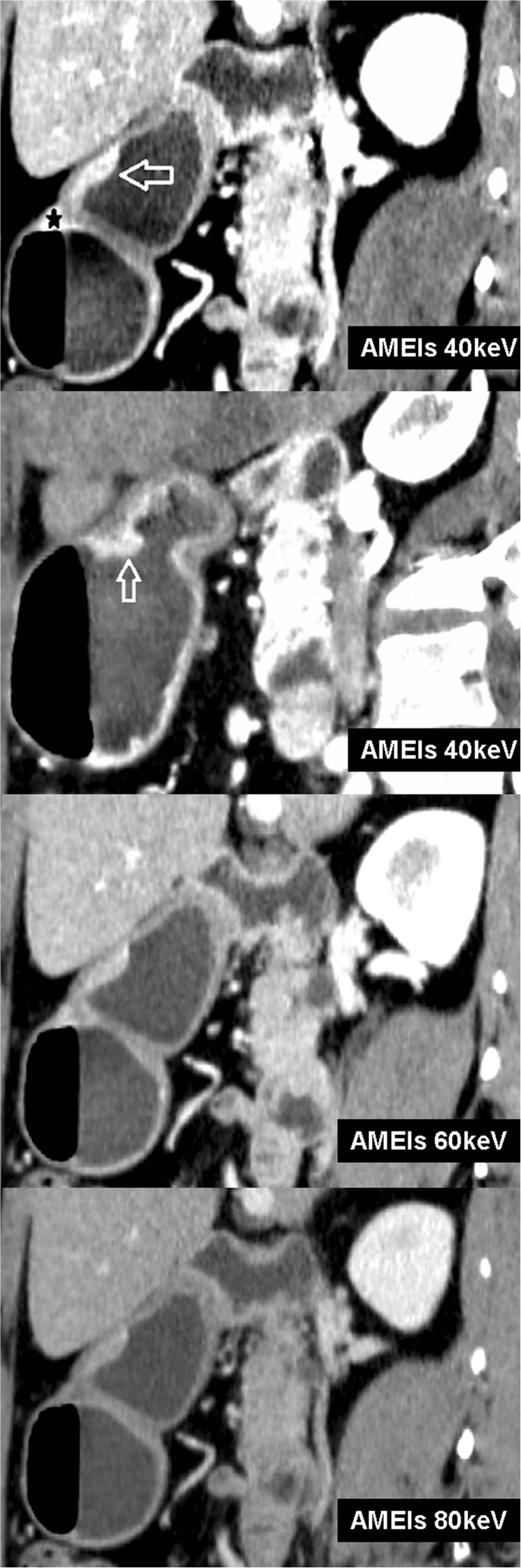
T1b cancer (60 yrs, female) in AM40, 60, 80 keV. AM40 keV sagittal image shows well-enhancing mucosal thickening (arrow) with an intact low-attenuation-strip outer layer in the gastric antrum and strong enhancement in the gastric angle (★) in the portal phase. AM40 keV oblique sagittal shows well-enhancing mucosal thickening (*arrow*) in the arterial phase in the gastric antrum; findings in these two reconstructive images suggest T1b cancer. The monoenergetic images not only increased the lesions’ CNR, but also highlighted existing artefact (★), which was caused by the air in the stomach with MPR. AM60 keV and AM80 keV show well-enhancing mucosal thickening with disruption of the low-density-stripe layer (greater than 50 % of the thickness). The tumour was identified as T2 cancer based on these images

**Fig. 6:**
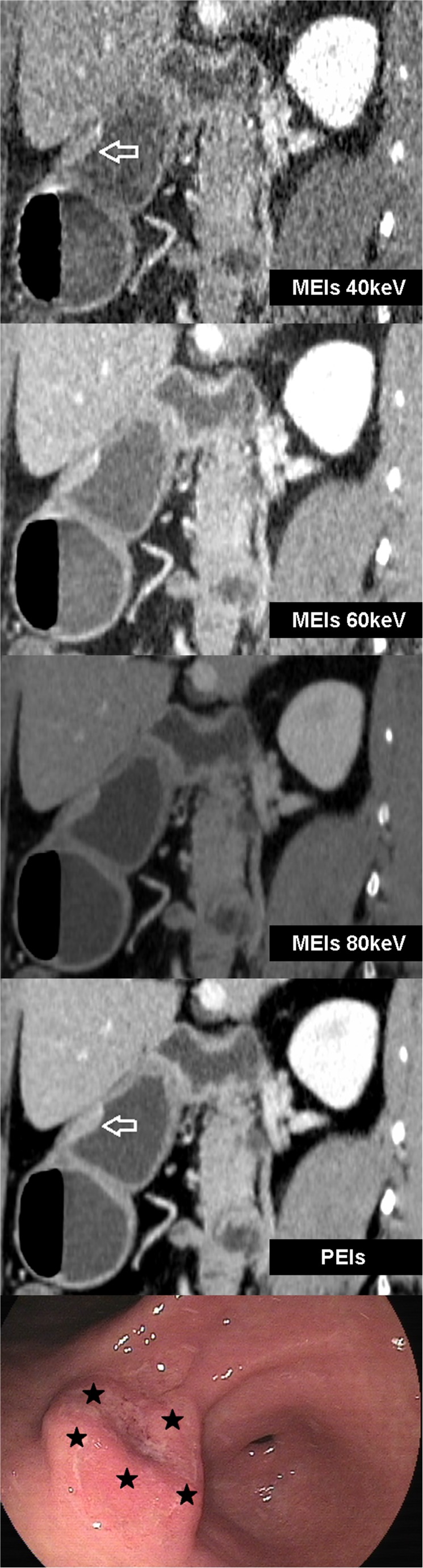
The same patient in Fig. 5 in M40, 60, 80 keV and PEIs. The discrimination between the enhancing gastric lesion and the outer layer is visually impossible on M40 keV and M60 keV, but a smooth outer margin of the outer layer or only a few small linear strandings in the perigastric fat plane are visualized. The tumour was identified as T3 cancer in these images. M80 keV and PEIs show well-enhancing mucosal thickening with disruption of the low-density-stripe layer (greater than 50 % of the thickness). The tumour was identified as T2 cancer basing on these images. Conventional gastroscopy image depicts a protruding lesion with the ulcer in the centre (★) in the gastric antrum

Twenty-five lesions from 31 patients were visible on AM40 keV data sets. Of these 25 lesions, five (16.1 %) lesions showed focal enhancement in AP, and 23 (74.2 %) showed strong enhancement in PP (with three lesions showing abnormal, strong enhancement in both AP and PP), with or without mural thickening. Twenty lesions were visible on PEIs. Among them, five lesions (16.1 %) showed focal enhancement in AP, and 18 lesions (58.1 %) showed strong enhancement in PP (with three lesions showing abnormal, strong enhancement in both AP and PP), with or without mural thickening. All visible lesions became indistinct in DEP. Five extra lesions, including four superficial type lesions and one excavated type lesion, were observed with AM40 keV, in contrast to the results observed with PEIs. All of these lesions showed strong enhancement of the inner hyperattenuating layer in PP and were invisible on PEIs (Figs. 3 and 4). Compared with AM40 keV, three more lesions, including two excavated type and one superficial-depressed-type lesions, were over-staged by PEIs (Figs. 5 and 6).

### Objective image analysis

**Fig. 7:**
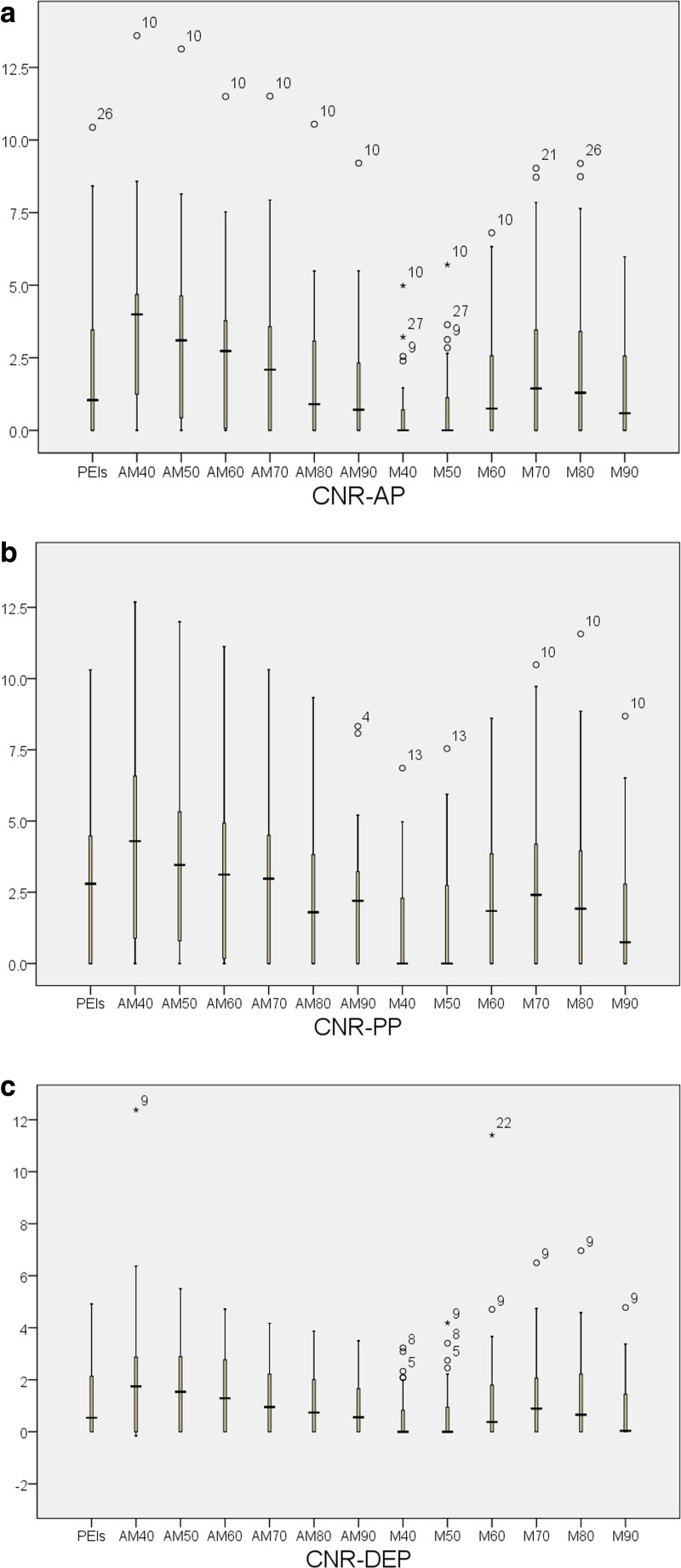
CNR results of all datasets in the arterial phase (CNR-AP)(a), portal phase (CNR-PP)(b) and delayed phase (CNR-DEP)(c)

The CNR results are listed in Fig. 7. The CNR-AP (CNR of the AP) and CNR-PP (CNR of the PP) of AM40 keV were significantly higher than for any other AMEIs, MEIs or PEIs (CNR-AP: 3.6 ± 3.0; CNR-PP: 4.4 ± 3.5; all *p* < 0.05). With regard to CNR-DEP (CNR of DEP), AM40 keV achieved the highest value, which was significantly different from those of the other AMEIs, MEIs, and PEIs (all *p* < 0.05) except for AM50 keV (*p* = 0.083). CNR-AP and CNR-PP for M40 keV and M50 keV were the lowest and were significantly different from those of other MEIs and PEIs (all *p* < 0.05). The CNR-AP and CNR-PP were significantly higher than CNR-DEP of AM40 keV (*p* = 0.034 and < 0.001, respectively). However, no significant difference was observed between CNR-AP and CNR-PP (*p* = 0.103). (Details are provided in “Supplementary Information”.)

### Subjective image analysis

Table 3 summarizes the subjective results for the MEIs, AMEIs and PEIs. AM40 keV gave a significantly higher gastric-specific diagnostic performance and visual sharpness compared with other AMEIs, MEIs and PEIs (all *p* < 0.05). With respect to image noise, PEIs had significant less noise compared with AMEIs and MEIs (all *p* < 0.05). M40 keV, M50 keV, and M60 keV had significantly higher image noise than did the other MEIs. The mean scores of image noise were acceptable for AM40 keV, AM50 keV, AM60 keV, AM70 keV, M70 keV and M80 keV. (Details are provided in “Supplementary Information”.)

**Table 3:** Subjective imaging analysis for PEIs, MEIs and AMEIs

group	Gastric-specific diagnostic performance	Image noise	Visual sharpness
PEIs	2.8 ± 0.7	2.0 ± 0.0	3.00 ± 0.0
AM40 keV	4.1 ± 0.7	2.9 ± 0.5	4.2 ± 0.4
AM50 keV	3.9 ± 0.8	2.9 ± 0.5	4.0 ± 0.4
AM60 keV	3.3 ± 0.9	2.5 ± 0.5	3.4 ± 0.5
AM70 keV	2.9 ± 0.8	2.3 ± 0.4	3.0 ± 0.0
AM80 keV	2.7 ± 0.8	2.1 ± 0.4	2.9 ± 0.3
AM90 keV	2.6 ± 0.9	2.1 ± 0.3	2.9 ± 0.3
M40 keV	1.6 ± 0.7	3.9 ± 0.4	1.9 ± 0.3
M50 keV	1.9 ± 0.8	3.7 ± 0.4	2.0 ± 0.3
M60 keV	2.5 ± 0.6	3.1 ± 0.3	2.6 ± 0.6
M70 keV	2.9 ± 0.7	2.7 ± 0.5	2.9 ± 0.3
M80 keV	2.9 ± 0.7	2.1 ± 0.3	2.9 ± 0.3
M90 keV	2.7 ± 0.6	2.1 ± 0.3	3.0 ± 0.0

### Inter-observer agreement

There is disagreement between two reviewers for the independent readings. The weighted *k* values of the visibility and over-staging were 0.806 and 0.734 (both *p* < 0.001), respectively. There was excellent inter-observer agreement with respect to the subjective image quality (*k* = 0.906 for gastric-specific diagnostic confidence, *k* = 0.922 for visual sharpness, and *k* = 0.891 for image noise, respectively) (all *p* < 0.001).

## Discussion

A previous study demonstrated that MEIs at 70 keV provided subjectively improved image qualities compared with PEIs in the evaluation of hepatic metastases [23], and that MEIs at 100 keV could significantly reduce dark-band-like artefacts, making it possible to evaluate the condition of bone-encircling dental implant bodies [24]. Nevertheless, few studies to date have investigated the application of MEIs or AMEIs to the stomach. Our study used the Mono+ algorithm to increase the CNR and decrease image noise at a low keV, and the results indicated that AM40 keV had the highest overall score: it resulted in significantly better visibility than M40 keV, M50 keV and M60 keV and showed a statistically significant lower stage migration than PEIs; it also had the highest CNR-AP, CNR-PP, and CNR-DEP, consistent with the gastric-specific diagnostic performance and visual sharpness results.

Although MEIs provides several benefits, such as increased signal of contrast agent and the possibility to reduce beam hardening, it carries the main drawback of a substantial increase in image noise at lower keVs. Thus, the gain in CNR with monoenergetic imaging, compared with a PEIs, or a single-energy scan at optimal kV is limited. To obtain better CNR results, a frequency-based recombination of the low-keV images (which contain high iodine contrast) and medium energies (typically approximately 70 keV, which received superior noise properties) was performed to combine the benefits of both stacks—the improved contrast and low noise [25]. Grant et al. investigated different image sets of phantoms to assess MEIs and AMEIs. Their results found out that the Mono+ algorithm provides the optimum iodine CNR at the lowest energy level of 40 keV [25]. As applied in our study, Mono+ increased the visibility of EGC with AM40 keV to 80.7 % on 2D and MPR images.

EGC, a hypervascular neoplasm [11], is often detected as areas of prominent contrast enhancement without mural thickening [26, 27]. Further, most EGCs are often not detected on PEIs because of the insufficient enhancement of focal lesions compared with the normal surrounding stomach walls. The incidence of EGCs with intense focal enhancement was 47 % [28]. In our PEIs data sets, 58.1 % of the lesions showed focal enhancement in PP, whereas 74.2 % of the lesions showed focal enhancement in the AM40 keV images. This result could be attributed to two factors: the scan protocol and the Mono+ algorithm. Because we used test bolus technique to individualize the scan delay time and achieve optimal contrast opacify [29], the PP, which corresponds to the arterial or mucosal phase scan time of previous reports [26], more accurately displayed the gastric mucosa for each patient, resulting in better detection of abnormal mucosal changes.

Because of the ability of the Mono+ algorithm to increase the lesion CNR, more lesions were visible on AM40 keV. Our AM40 keV data sets were able to reveal 25 lesions in 31 patients. Although the visibility of EGC in a CT scan is strongly influenced by its morphological type and elevated-type EGCs are easier to detect than superficial or depressed-type cancers, five extra lesions, including four superficial-type lesions and one excavated-type lesion, were shown in AM40 keV images compared with PEIs. All of these lesions showed strong enhancement of the inner hyperattenuating layer in PP, which were invisible on PEIs. AM40 keV has higher CNR-APs and CNR-PPs than any other AMEIs, MEIs and PEIs. Because our CNR results were calculated using the contrast between gastric lesions and normal gastric wall, a higher CNR may lead to a better image of the lesions. Thus, we believe that EGCs that are invisible (i.e., superficial-type or excavated-type) using conventional CT could be depicted more clearly using AM40 keV. The same applies to the decreased stage migration in AM40 keV images compared to PEIs.

The decreased stage migration in AM40 keV images compared with PEIs, which over-staged an additional three lesions, including two excavated-type lesions and one superficial-depressed-type lesion, indicating a clearer depiction of EGCs. The most reliable diagnostic criterion for differentiating EGC from AGC at MDCT is a good visualization of the low-attenuation-strip outer layer of the gastric wall [11]; however, defining the depth of tumour invasion in cases of T1b was usually difficult because the low-attenuation-strip outer layer was obscured. This might be due to the thinning of the gastric wall related to distension or inflammation or oedema in the muscular layer beneath the primary lesion. Therefore, we often need to distinguish T1b tumours from T2 or even T3 tumours. Our study revealed that AM40 keV could stage EGC more correctly than PEIs (20 vs. 12), owing to the clear depiction of the gastric wall using the Mono+ algorithm, which indicated a better discrimination of T1b tumours from more advance tumours.

Conventional lower-energy techniques result in increased image noise via increased quantum mottle. Consistent with this finding, M40 keV, M50 keV and M60 keV showed statistically higher image noise score, which affected the diagnostic interpretation. Using the Mono+ algorithm, the noise in the AM40 keV images was significantly decreased compared with M40 keV, M50 keV and M60 keV and did not affect the diagnostic interpretation. In short, dual-energy scan with dual-source CT of the stomach is feasible in routine clinical practice, and AMEIs at 40 keV can decrease the stage migration of EGC.

One limitation of our study was its small number of patients, which introduces the potential for unintended biases. Further research using a larger patient population is necessary. In addition, even though the principle of the design in this study is randomization and double-blind, there are still unintentional biases in the study involving subjective judgements. Furthermore, we only compared the 2D axial and MPR images for three types of image data sets. The detection and stage results of 3D reconstruction such as virtual endoscopy based on AMEIs data sets could be investigated in a future study.

In conclusion, 2D advanced image-based calculated virtual 40 keV images with MPR significantly increase the CNR of EGC, leading to significantly decreased stage migration of EGC.

## Electronic supplementary material

Below is the link to the electronic supplementary material.

ESM 1 (DOC 177 kb)

## Abbreviations

EGC: Early gastric cancer
MPR: Multiplanar reconstruction
2D: Two-dimensional
3D: Three-dimensional
kV: Kilovoltage
keV: Kiloelectron volt
PEIs: Polyenergetic images
MEIs: Monoenergetic images
AMEIs: Advanced monoenergetic images
AP: Arterial phase
PP: Portal venous phase
DEP: Delayed phase
