# Supplementary material for: Decreased stage migration rate of early gastric cancer with a new reconstruction algorithm using dual-energy CT images: a preliminary study
Source: Eur Radiol. 2016 Jun 8;27(2):671–80. doi: 10.1007/s00330-016-4442-z (PMC5209438; doi:10.1007/s00330-016-4442-z)
Supplement: Supplementary file 1 — (DOC 177 kb) [file 330_2016_4442_MOESM1_ESM.doc]

**Decreased stage migration rate of early gastric cancer with a new reconstruction algorithm using dual-energy CT images: a preliminary study**

Table 1 Comparison results for visibility*

|  | PEIs | AM40 | AM50 | AM60 | AM70 | AM80 | AM90 | M40 | M50 | M60 | M70 | M80 | M90 |
| --- | --- | --- | --- | --- | --- | --- | --- | --- | --- | --- | --- | --- | --- |
| PEIs | – | 0.146 | 0.231 | 0.351 | 0.590 | 0.756 | 0.756 | 0.025 | 0.045 | 0.683 | 0.756 | 0.756 | 0.756 |
| AM40 | – | – | 0.756 | 0.590 | 0.351 | 0.231 | 0.231 | 0.008 | 0.008 | 0.045 | 0.231 | 0.231 | 0.231 |
| AM50 | – | – | – | 0.756 | 0.590 | 0.351 | 0.351 | 0.008 | 0.012 | 0.080 | 0.351 | 0.351 | 0.351 |
| AM60 | – | – | – | – | 0.756 | 0.590 | 0.590 | 0.012 | 0.017 | 0.231 | 0.683 | 0.683 | 0.683 |
| AM70 | – | – | – | – | – | 0.756 | 0.756 | 0.017 | 0.018 | 0.351 | 0.756 | 0.756 | 0.756 |
| AM80 | – | – | – | – | – | – | 0.756 | 0.018 | 0.025 | 0.492 | 0.756 | 0.756 | 0.756 |
| AM90 | – | – | – | – | – | – | – | 0.018 | 0.025 | 0.492 | 0.756 | 0.756 | 0.756 |
| M40 | – | – | – | – | – | – | – | – | 0.756 | 0.080 | 0.018 | 0.018 | 0.018 |
| M50 | – | – | – | – | – | – | – | – | – | 0.146 | 0.025 | 0.025 | 0.025 |
| M60 | – | – | – | – | – | – | – | – | – | – | 0.351 | 0.351 | 0.492 |
| M70 | – | – | – | – | – | – | – | – | – | – | – | 0.756 | 0.756 |
| M80 | – | – | – | – | – | – | – | – | – | – | – | – | 0.756 |
| M90 | – | – | – | – | – | – | – | – | – | – | – | – | – |

*: Data are presented as the adjusted *p* value of McNemar test between two groups

Table 2 Comparison results for stage migration*

|  | PEIs | AM40 | AM50 | AM60 | AM70 | AM80 | AM90 | M40 | M50 | M60 | M70 | M80 | M90 |
| --- | --- | --- | --- | --- | --- | --- | --- | --- | --- | --- | --- | --- | --- |
| PEIs | – | 0.026 | 0.083 | 0.250 | 0.443 | 0.443 | 0.443 | a | a | 0.039 | 0.443 | 0.250 | 0.250 |
| AM40 | – | – | 0.250 | 0.083 | 0.064 | 0.010 | 0.010 | a | a | 0.002 | 0.010 | 0.010 | 0.010 |
| AM50 | – | – | – | 0.443 | 0.250 | 0.039 | 0.039 | a | a | 0.008 | 0.039 | 0.026 | 0.026 |
| AM60 | – | – | – | – | 0.709 | 0.083 | 0.083 | a | a | 0.010 | 0.083 | 0.064 | 0.064 |
| AM70 | – | – | – | – | – | 0.143 | 0.143 | a | a | 0.017 | 0.143 | 0.083 | 0.083 |
| AM80 | – | – | – | – | – | – | 0.709 | a | a | 0.083 | 0.709 | 0.709 | 0.709 |
| AM90 | – | – | – | – | – | – | – | a | a | 0.083 | 0.709 | 0.709 | 0.709 |
| M40 | – | – | – | – | – | – | – | – | a | a | a | a | a |
| M50 | – | – | – | – | – | – | – | – | – | a | a | a | a |
| M60 | – | – | – | – | – | – | – | – | – | – | 0.083 | 0.143 | 0.143 |
| M70 | – | – | – | – | – | – | – | – | – | – | – | 0.709 | 0.709 |
| M80 | – | – | – | – | – | – | – | – | – | – | – | – | 0.709 |
| M90 | – | – | – | – | – | – | – | – | – | – | – | – | – |

*: Data are presented as the adjusted *p* value of McNemar test between two groups

a: The results of M40 and M50 are too small to compare

Table 3 Comparison results for CNR-AP*

|  | PEIs | AM40 | AM50 | AM60 | AM70 | AM80 | AM90 | M40 | M50 | M60 | M70 | M80 | M90 |
| --- | --- | --- | --- | --- | --- | --- | --- | --- | --- | --- | --- | --- | --- |
| PEIs | – | 0.001 | 0.005 | 0.034 | 0.098 | 0.033  - | 0.005 | 0.001 | 0.001 | 0.007 | 0.180 | 0.152 | 0.004 |
| AM40 | – | – | 0.002 | 0.000 | 0.000 | 0.000 | 0.000 | 0.000 | 0.000 | 0.000 | 0.001 | 0.002 | 0.000 |
| AM50 | – | – | – | 0.007 | 0.002 | 0.000 | 0.000 | 0.000 | 0.000 | 0.000 | 0.006 | 0.011 | 0.000 |
| AM60 | – | – | – | – | 0.034 | 0.001 | 0.000 | 0.000 | 0.000 | 0.001 | 0.041 | 0.063 | 0.001 |
| AM70 | – | – | – | – | – | 0.003 | 0.001 | 0.000 | 0.000 | 0.002 | 0.095 | 0.136 | 0.003 |
| AM80 | – | – | – | – | – | – | 0.007 | 0.000 | 0.001 | 0.044 | 0.029 | 0.018 | 0.053 |
| AM90 | – | – | – | – | – | – | – | 0.000 | 0.002 | 0.165 | 0.006 | 0.004 | 0.144 |
| M40 | – | – | – | – | – | – | – | – | 0.007 | 0.001 | 0.000 | 0.000 | 0.001 |
| M50 | – | – | – | – | – | – | – | – | – | 0.004 | 0.001 | 0.001 | 0.003 |
| M60 | – | – | – | – | – | – | – | – | – | – | 0.000 | 0.001 | 0.176 |
| M70 | – | – | – | – | – | – | – | – | – | – | – | 0.061 | 0.015 |
| M80 | – | – | – | – | – | – | – | – | – | – | – | – | 0.007 |
| M90 | – | – | – | – | – | – | – | – | – | – | – | – | – |

*: Data are presented as the adjusted *p* value of paired *t*-test between two groups

Table 4 Comparison results for CNR-PP*

|  | PEIs | AM40 | AM50 | AM60 | AM70 | AM80 | AM90 | M40 | M50 | M60 | M70 | M80 | M90 |
| --- | --- | --- | --- | --- | --- | --- | --- | --- | --- | --- | --- | --- | --- |
| PEIs | – | 0.000 | 0.002 | 0.024 | 0.127 | 0.004 | 0.004 | 0.000 | 0.000 | 0.002 | 0.064 | 0.044 | 0.000 |
| AM40 | – | – | 0.000 | 0.000 | 0.000 | 0.000 | 0.000 | 0.000 | 0.000 | 0.000 | 0.000 | 0.000 | 0.000 |
| AM50 | – | – | – | 0.000 | 0.000 | 0.000 | 0.000 | 0.000 | 0.000 | 0.000 | 0.002 | 0.001 | 0.000 |
| AM60 | – | – | – | – | 0.000 | 0.000 | 0.000 | 0.000 | 0.000 | 0.001 | 0.015 | 0.007 | 0.000 |
| AM70 | – | – | – | – | – | 0.001 | 0.000 | 0.000 | 0.000 | 0.007 | 0.077 | 0.050 | 0.000 |
| AM80 | – | – | – | – | – | – | 0.019 | 0.001 | 0.002 | 0.077 | 0.017 | 0.027 | 0.006 |
| AM90 | – | – | – | – | – | – | – | 0.002 | 0.007 | 0.123 | 0.011 | 0.016 | 0.017 |
| M40 | – | – | – | – | – | – | – | – | 0.005 | 0.000 | 0.000 | 0.000 | 0.011 |
| M50 | – | – | – | – | – | – | – | – | – | 0.001 | 0.000 | 0.001 | 0.038 |
| M60 | – | – | – | – | – | – | – | – | – | – | 0.000 | 0.010 | 0.032 |
| M70 | – | – | – | – | – | – | – | – | – | – | – | 0.050 | 0.001 |
| M80 | – | – | – | – | – | – | – | – | – | – | – | – | 0.001 |
| M90 | – | – | – | – | – | – | – | – | – | – | – | – | – |

*: Data are presented as the adjusted *p* value of paired *t*-test between two groups

Table 5 Comparison results for CNR-DEP*

|  | PEIs | AM40 | AM50 | AM60 | AM70 | AM80 | AM90 | M40 | M50 | M60 | M70 | M80 | M90 |
| --- | --- | --- | --- | --- | --- | --- | --- | --- | --- | --- | --- | --- | --- |
| PEIs | – | 0.010 | 0.015 | 0.044 | 0.277 | 0.211 | 0.066 | 0.007 | 0.016 | 0.279 | 0.130 | 0.191 | 0.043 |
| AM40 | – | – | 0.083 | 0.026 | 0.008 | 0.002 | 0.002 | 0.001 | 0.001 | 0.041 | 0.007 | 0.004 | 0.002 |
| AM50 | – | – | – | 0.055 | 0.017 | 0.002 | 0.001 | 0.001 | 0.001 | 0.133 | 0.064 | 0.043 | 0.002 |
| AM60 | – | – | – | – | 0.048 | 0.007 | 0.002 | 0.001 | 0.001 | 0.245 | 0.211 | 0.150 | 0.008 |
| AM70 | – | – | – | – | – | 0.043 | 0.008 | 0.001 | 0.003 | 0.300 | 0.185 | 0.282 | 0.043 |
| AM80 | – | – | – | – | – | – | 0.007 | 0.007 | 0.019 | 0.187 | 0.021 | 0.070 | 0.138 |
| AM90 | – | – | – | – | – | – | – | 0.021 | 0.066 | 0.130 | 0.008 | 0.022 | 0.282 |
| M40 | – | – | – | – | – | – | – | – | 0.016 | 0.033 | 0.001 | 0.003 | 0.066 |
| M50 | – | – | – | – | – | – | – | – | – | 0.048 | 0.002 | 0.004 | 0.134 |
| M60 | – | – | – | – | – | – | – | – | – | – | 0.333 | 0.357 | 0.130 |
| M70 | – | – | – | – | – | – | – | – | – | – | – | 0.187 | 0.021 |
| M80 | – | – | – | – | – | – | – | – | – | – | – | – | 0.015 |
| M90 | – | – | – | – | – | – | – | – | – | – | – | – | – |

*: Data are presented as the adjusted *p* value of paired *t*-test between two groups

**Table 6 Comparison results for gastric-specific diagnostic** performance*

|  | PEIs | AM40 | AM50 | AM60 | AM70 | AM80 | AM90 | M40 | M50 | M60 | M70 | M80 | M90 |
| --- | --- | --- | --- | --- | --- | --- | --- | --- | --- | --- | --- | --- | --- |
| PEIs | – | 0.000 | 0.000 | 0.003 | 0.093 | 0.053 | 0.015 | 0.000 | 0.000 | 0.001 | 0.146 | 0.141 | 0.041 |
| AM40 | – | – | 0.003 | 0.000 | 0.000 | 0.000 | 0.000 | 0.000 | 0.000 | 0.000 | 0.000 | 0.000 | 0.000 |
| AM50 | – | – | – | 0.000 | 0.000 | 0.000 | 0.000 | 0.000 | 0.000 | 0.000 | 0.000 | 0.000 | 0.000 |
| AM60 | – | – | – | – | 0.000 | 0.000 | 0.000 | 0.000 | 0.000 | 0.000 | 0.004 | 0.004 | 0.001 |
| AM70 | – | – | – | – | – | 0.005 | 0.002 | 0.000 | 0.000 | 0.002 | 0.122 | 0.122 | 0.031 |
| AM80 | – | – | – | – | – | – | 0.024 | 0.000 | 0.000 | 0.031 | 0.044 | 0.044 | 0.152 |
| AM90 | – | – | – | – | – | – | – | 0.000 | 0.001 | 0.139 | 0.013 | 0.013 | 0.047 |
| M40 | – | – | – | – | – | – | – | – | 0.006 | 0.000 | 0.000 | 0.000 | 0.000 |
| M50 | – | – | – | – | – | – | – | – | – | 0.000 | 0.000 | 0.000 | 0.000 |
| M60 | – | – | – | – | – | – | – | – | – | – | 0.000 | 0.001 | 0.005 |
| M70 | – | – | – | – | – | – | – | – | – | – | – | 0.192 | 0.024 |
| M80 | – | – | – | – | – | – | – | – | – | – | – | – | 0.024 |
| M90 | – | – | – | – | – | – | – | – | – | – | – | – | – |

*: Data are presented as the adjusted *p* value of Wilcoxon signed rank test between two groups

Table 7 Comparison results for image noise*

|  | PEIs | AM40 | AM50 | AM60 | AM70 | AM80 | AM90 | M40 | M50 | M60 | M70 | M80 | M90 |
| --- | --- | --- | --- | --- | --- | --- | --- | --- | --- | --- | --- | --- | --- |
| PEIs | – | 0.000 | 0.000 | 0.000 | 0.001 | 0.028 | 0.015 | 0.000 | 0.000 | 0.000 | 0.000 | 0.009 | 0.015 |
| AM40 | – | – | 0.067 | 0.000 | 0.000 | 0.000 | 0.000 | 0.000 | 0.000 | 0.033 | 0.008 | 0.000 | 0.000 |
| AM50 | – | – | – | 0.001 | 0.000 | 0.000 | 0.000 | 0.000 | 0.000 | 0.007 | 0.013 | 0.000 | 0.000 |
| AM60 | – | – | – | – | 0.004 | 0.000 | 0.000 | 0.000 | 0.000 | 0.000 | 0.058 | 0.001 | 0.000 |
| AM70 | – | – | – | – | – | 0.003 | 0.011 | 0.000 | 0.000 | 0.000 | 0.002 | 0.043 | 0.024 |
| AM80 | – | – | – | – | – | – | 0.054 | 0.000 | 0.000 | 0.000 | 0.000 | 0.067 | 0.103 |
| AM90 | – | – | – | – | – | – | – | 0.000 | 0.000 | 0.000 | 0.000 | 0.110 | 0.154 |
| M40 | – | – | – | – | – | – | – | – | 0.007 | 0.000 | 0.000 | 0.000 | 0.000 |
| M50 | – | – | – | – | – | – | – | – | – | 0.000 | 0.000 | 0.000 | 0.000 |
| M60 | – | – | – | – | – | – | – | – | – | – | 0.000 | 0.000 | 0.000 |
| M70 | – | – | – | – | – | – | – | – | – | – | – | 0.000 | 0.00 |
| M80 | – | – | – | – | – | – | – | – | – | – | – | – | 0.103 |
| M90 | – | – | – | – | – | – | – | – | – | – | – | – | – |

*: Data are presented as adjusted *p* value of Wilcoxon signed rank test between two groups

Table 8 Comparison results for Visual sharpness*

|  | PEIs | AM40 | AM50 | AM60 | AM70 | AM80 | AM90 | M40 | M50 | M60 | M70 | M80 | M90 |
| --- | --- | --- | --- | --- | --- | --- | --- | --- | --- | --- | --- | --- | --- |
| PEIs | – | 0.000 | 0.000 | 0.000 | 0.231 | 0.041 | 0.024 | 0.000 | 0.000 | 0.000 | 0.024 | 0.041 | 0.231 |
| AM40 | – | – | 0.015 | 0.000 | 0.000 | 0.000 | 0.000 | 0.000 | 0.000 | 0.000 | 0.000 | 0.000 | 0.000 |
| AM50 | – | – | – | 0.000 | 0.000 | 0.000 | 0.000 | 0.000 | 0.000 | 0.000 | 0.000 | 0.000 | 0.000 |
| AM60 | – | – | – | – | 0.000 | 0.000 | 0.000 | 0.000 | 0.000 | 0.000 | 0.000 | 0.000 | 0.000 |
| AM70 | – | – | – | – | – | 0.041 | 0.024 | 0.000 | 0.000 | 0.000 | 0.024 | 0.041 | 0.231 |
| AM80 | – | – | – | – | – | – | 0.082 | 0.000 | 0.000 | 0.002 | 0.162 | 0.231 | 0.041 |
| AM90 | – | – | – | – | – | – | – | 0.000 | 0.000 | 0.004 | 0.231 | 0.162 | 0.024 |
| M40 | – | – | – | – | – | – | – | – | 0.024 | 0.000 | 0.000 | 0.000 | 0.000 |
| M50 | – | – | – | – | – | – | – | – | – | 0.000 | 0.000 | 0.000 | 0.000 |
| M60 | – | – | – | – | – | – | – | – | – | – | 0.002 | 0.000 | 0.000 |
| M70 | – | – | – | – | – | – | – | – | – | – | – | 0.143 | 0.024 |
| M80 | – | – | – | – | – | – | – | – | – | – | – | – | 0.041 |
| M90 | – | – | – | – | – | – | – | – | – | – | – | – | – |

*: Data are presented as adjusted *p* value of Wilcoxon signed rank test between two groups
